# Alcohol use among university students in Sweden measured by an electronic screening instrument

**DOI:** 10.1186/1471-2458-9-229

**Published:** 2009-07-13

**Authors:** Agneta Andersson, Ann-Britt Wiréhn, Christina Ölvander, Diana Stark Ekman, Preben Bendtsen

**Affiliations:** 1 Division of Social Medicine and Public Health Science, Department of Medical and Health Sciences (IMH), Linköping University, SE-581 83, Linköping, Sweden; 2 Local Health Care Research and Development Unit, County Council in Östergötland, SE-581 85 Linköping, Sweden; 3 Division of Social Medicine, Department of Public Health Sciences, Karolinska Institutet, Stockholm, SE-171 77 Stockholm, Sweden

## Abstract

**Background:**

Electronic-based alcohol screening and brief interventions for university students with problem drinking behaviours forms an important means by which to identify risky drinkers.

**Methods:**

In this study an e-SBI project was implemented to assess drinking patterns, and to provide personalised feedback about alcohol consumption and related health problems, to students in a Swedish university. In this study, third semester university students (n = 2858) from all faculties (colleges) at the University were invited to participate in e-SBI screenings. This study employed a randomised controlled trial, with respondents having a equal chance of being assigned to a limited, or full-feedback response.

**Results:**

The study shows that high risk drinkers tend to underestimate their own consumption compared to others, and that these high risk drinkers experience more negative consequences after alcohol intake, than other respondents. There was a strong belief, for both high- and low-risk drinkers, that alcohol helped celebrations be more festive. This study also confirms findings from other study locations that while males drank more than females in our study population; females reached the same peak alcohol blood concentrations as males.

**Conclusion:**

Obtaining clear and current information on drinking patterns demonstrated by university students can help public health officials, university administration, and local health care providers develop appropriate prevention and treatment strategies.

## Background

### Electronic-based alcohol screening and brief interventions for university students with problem drinking behaviours

Screening for alcohol intake in university student populations is an important first step to identify risky drinkers, and institute more effective services for this group, as university students represent a group at high risk for excessive alcohol consumption. Excessive alcohol consumption in university student populations has been linked to multiple health problems, including drunk driving, elevated risk for injuries, and development of health problems over time [1-4]. Several recent studies have assessed the drinking patterns of Swedish university students and found that the consumption patterns by some university students in this country are at levels that are likely to cause problems, not only with health, but with academic performance. This increased consumption may already be present with students arrive at university: a study of Swedish freshmen studying in Luleå and Växjö found a generally high level of alcohol consumption in this group [5,3] surveyed the drinking patterns of 359 freshmen in Lund and found that student consumption patterns could roughly be divided into four categories-steady and high consumption, increasing consumption over time, decreasing consumption over time, and steady consumption. While 60% of students could be considered steady consumers, about 25% of the population fell into steady and high, or increasing consumption patterns over time. This pattern is similar to those seen in other university settings in Sweden and the US, according to the authors. Recent studies assessing alcohol intake by university students have found gender differences [6,7]. A study comparing consumption patterns of Swedish and US university students, conducted by Ståhlbrandt et al. (2008) [4] found that alcohol expectancies, that is, self-reported measures anticipation experiences associated with alcohol use, was higher among Swedish male students than US male students. Overall, though, the study found that the overall pattern of relationships between alcohol intake and predictors was comparable in both groups, suggesting that findings in similar studies could be generalisable.

A meta-analysis of drinking patterns in university students found that these patterns were more or less comparable for groups in North America, Europe, Australasia and South America, with lower consumption patterns seen in Southeast Asia and Africa [1]. This study found that intermediary survey methods to measure alcohol intake, and deliver tailored advice, based on personal intake levels, were acceptable to students, not only in Sweden, but also in New Zealand. Furthermore, use of intermediary survey methods can provide generalisable results, at least for the student population of interest, those who engage in more frequent or heavy drinking. Cranford et al. (2008) [8] conducted a voluntary web survey and then conducted a follow up telephone interview with non-responders. Their study found that there were no gender differences between the responder and non-responder groups, but that the non-responder group had less overall alcohol intake, compared to survey responders, and the first group spent more time preparing for classes than responders. This study found that the most common reasons for nonresponse to the web survey included "too busy" (45.7%), "not interested" (18.1%) and "forgot to complete the survey" (18.1%).

This use of intermediary survey methods has resulted in multiple ongoing studies of alcohol use in student populations. For example, a recent postal survey of university students reported that 96% of respondents had consumed alcohol in the 12 months prior to completing the survey, and 33% reported binge drinking twice a month or more often [9]. Electronic, or computerized, screenings to measure alcohol intake and alcohol-related behaviours, partnered with immediate brief interventions (e-SBIs) have been well-accepted by university students in multiple countries [10-12]. Advantages of e-SBIs for students include round-the-clock access to anonymous services; little, if any required interaction with clinicians; and familiarity of computer-based surveys [13]. The e-SBI approach allows a research advantage, as it is an easily-delivered survey method to screen large numbers of students at limited cost [14].

The results of student surveys can provide feedback to respondents on 'normal' alcohol consumption patterns, with information aimed particularly to students who demonstrate unhealthy alcohol use habits. While personalised feedback is enhanced with use of e-SBI, little research exists that identifies the best way to present such information to various student populations in a way that results in changing drinking behaviours. More research is needed on the amount of feedback and follow up contact, and the optimum time frame in which to provide feedback, that promotes changes in respondents' drinking behaviours.

### Purpose of current study

In this study an e-SBI project was implemented to assess drinking patterns, and to provide personalised feedback about alcohol consumption and related health problems to students in a Swedish university.

Study aims were: 1. to analyse drinking habits including self-reported weekly consumption, heavy-episodic drinking, HED, and estimated peak blood alcohol concentration, EBAC; 2. to analyse respondents' perceptions of their own alcohol consumption in relation to consumption by other students, (i.e. normative beliefs); and, 3. to analyse reports of beliefs about alcohol use and negative experiences related to alcohol consumption, either by the individual student respondents or other students.

The study was approved by the ethics committee in Linkoping University, DNR 141-07. On the first web-page in the test, information about the study was presented and the student was also informed that by completing the test he or she had entered the study.

## Methods

### Study Population

The city of Linköping has a population of about 140 000 people and is situated in the southeast of Sweden with about 40 000 other residents living within the municipality. More than 25 000 students were registered at Linköping University (LiU) in 2007, which ranks it among the most-populated universities in Sweden. In this study, third semester university students (n = 2858) from all faculties (colleges) at the University were invited to participate in e-SBI screenings from fall 2007 through spring 2008. The target population was selected on the assumption that students in their third semester have more established drinking patterns compared to first and second semester students.

### Participants and recruitment procedure

In the beginning of October 2007 e-mails were distributed to all third-semester LiU students using their university e-mail addresses, inviting these students to test alcohol consumption patterns as well as participate in a study focusing on university students' drinking habits. Each message included a one-time-use-only hyperlink to the test. By following the hyperlink, the student's web-browser opened to a site containing an e-SBI questionnaire. The students' e-mail addresses served as unique identifiers. At the end of the test the participants were asked to type their e-mail address as a personal identifier, thus (actively) agreeing to participate in follow-up e-SBIs at 3 and 6 months respectively. One week after the first e-mail a reminder, including the same information (and a hyperlink) as the first, was sent to all participants including an apology for those who had already responded to the first request. The mailing process was carried through by UNIT, the department of IT support at Linkoping University.

Response to the e-SBI survey by LiU's third term students was almost 46%, varying by academic discipline. (See Table 1.) Of the 1308 students who responded, 11 had missing data and were excluded from the analysis thus leaving 1297 complete responses on which the analysis was made.

**Table 1:** Number of students receiving the first e-mail (n) and response rate distributed by academic discipline

University section	n (%)	Response % (F %/M %)
Faculty of arts and science	915 (32)	57 (61/39)
Institute of technology	972 (34)	45 (32/68)
Institute of education	657 (23)	24 (72/28)
Faculty of health sciences	314 (11)	57 (76/24)
Total	2858 (100)	46 (55/45)

### Access to the instrument

The alcohol survey instrument was created using ASP.NET and the freestanding programmes in Microsoft Visual Studio 2005. The questionnaire and database were hosted by a web-service provider. In the e-SBI survey, responses were collected using check-boxes and dropdown lists, depending on the type of question. It was possible for the respondent to exit the test at any time. The result of the test was not saved in the database until the last "continue" button was clicked. Thereafter access to the test was blocked for that respondent. The test (in Swedish) can be viewed at .

### Items measured in the e-SBI

In order to estimate alcohol consumption by respondents, the test measured weekly consumption and heavy-episodic drinking (HED) using standard glasses as a base measurement (one standard glass = 12 grams of alcohol). Respondents estimated weekly consumption by identifying how many standard glasses of alcohol they had consumed each day of the previous week. HED was considered to be 4 drinks or more for women and 5 drinks or more for men, on one occasion.

Respondents were asked to identify the most standard glasses consumed during one occasion in the past three months. For this peak consumption occasion, respondents were asked about the number of hours this occasion lasted. Respondents were also asked about body weight.

In order to calculate estimated peak blood alcohol concentration (EBAC) a variation, including drinking period in hours, of the Widmark formula was used [15]. The formula is:



where 0.806 is a constant for body water in the blood (mean 80.6%), SD is the number of standard drinks containing 10 grams of ethanol, 1.2 is a factor in order to convert the amount in grams to Swedish standards set by The Swedish National Institute of Public Health, BW is a body water constant (0.58 for men and 0.49 for women), Wt is body weight (kilogram), MR is the metabolism constant (0.017), DP is the drinking period in hours and 10 converts the result to permillage of alcohol.

Respondents were asked to describe how often they believed others drank, and how many standard drinks per occasion these others drank. "Others" were defined in three categories: your closest friends, an average student at Linköping University and an average Swedish person. The last two categories were self-adjusting based on the sex and age stated by the respondent, i.e. if the respondent was a 22 year old female she was asked "How much do you think that an average *21–25 year old female *student at Linkoping University drinks?"

Respondents were asked to report negative consequences of their alcohol consumption habits in regards to their studies, personal economy, and relations to family and friends. Students were also asked about physical injury and being exposed to violence, or the threat of violence.

The e-SBI items included statements about the desirability of alcohol consumption and social consequences of alcohol consumption, with which the respondents were asked to agree or disagree. Respondents were also asked about their motivation to change alcohol drinking habits early in the test and immediately after they had completed the test.

As students completed each e-SBI, a printable web page with the personalised feedback was presented. Upon completion of the survey, the respondent was thanked for participating in the test. The page also included a hyperlink to The Student Health Care Centre at Linkoping University for more information on problem drinking behaviours or treatment options.

### Personalised feedback

After surveys were completed, study participants were randomly assigned to either a control response with a minimal amount of feedback, consisting of three statements, or an intervention response with extensive feedback, consisting of up to 17 possible statements, dependant on respondents' answers.

The cut off for risky drinking for men was 15 or more drinks per week and/or 5 drinks or more at one occasion once per week or more. The corresponding cut off for women was 10 or more drinks per week and/or 4 drinks or more at one occasion once per week or more. However, in both feedback methods, weekly alcohol consumption, heavy episodic drinking and estimated blood alcohol content, permillage, were graded on three rectangular boxes constituting a horizontal scale with low health risk (green), extended health risk (yellow) and high health risk (red) for each consumption level. Readers will note that in this report, responses from students in low- and increased-risk groups are combined, creating a 'low-risk' category in order to facilitate comparisons with other studies.

The extensive feedback version provided to some respondents included information on the caloric value of the highest amount of alcohol consumed at one occasion by each respondent. Reference values set by the Swedish National Food Administration resulted in a standard of 7 kcal/gram alcohol [16]. In addition, information on the average number of calories needed per day by each respondent was provided, based on the age and sex stated by the respondent. In the extensive feedback responses, there was also normative feedback given regarding weekly alcohol consumption and HED. The results from a previous study [11] were used to calculate an estimate of weekly consumption and heavy episodic drinking by a typical student at Linköping University. The results were presented as two separate rectangular graphs, one for weekly consumption and one for HED including an explanatory text.

The extensive feedback version provided respondents with findings and advice on modifying their alcohol drinking behaviours-twelve possible responses were listed. Non-applicable advice was still readable, to allow the student to see what other possible responses might have been provided, had she or he described other drinking behaviours. Findings and advice included the following statements, among others; "According to your test results, you are in control of your alcohol use"; "It appears that your alcohol use is negatively affecting your life"; "Think about what you can do to reduce your alcohol consumption"; "Your answers indicate that you don't think you have an alcohol consumption problem, but your answers show that your drinking behaviours are high risk"; "Try to drink less than five standard servings of alcohol at any one occasion"; and, "You might consider replacing some drinks with alcohol-free alternatives".

Respondents who had extensive feedback were also provided illustrations of several different types of alcohol servings, to facilitate the interpretation of the applicable advice responses.

### Data processing

The data were extracted from a database placed at a web-hotel (Loopia) to an Excel file and thereafter to SPSS 15.0 where the statistical analyses were performed.

### Study design and statistical analyses

This study employed a randomised controlled trial design, with respondents having a equal chance of being assigned to a limited, or full-feedback response. The percentage of respondents having 4.3 4 to 5 drinks or more on one occasion, HED, are presented by gender for age groups 18–20, 21–25, 26–30, 31–35 and > 35 years. Weekly permillage alcohol consumption and EBAC are presented as mean (SD) as well as median (25 percentile, 75 percentile) values. Perception of drinking in comparison with other students is presented in numbers and percentage of consumption pattern (high versus low risk). Respondents' reported negative consequences due to alcohol consumption were analysed with logistic regression using consumption pattern (high versus low risk) as the dependent variable and responses to eight given statements by the student respondents as independent variables, one at a time. Odds ratios (OR) were presented with 95% confidence intervals (CIs). Adjustments were made by gender and academic discipline for each estimated OR. A significance level of 5% was used.

## Results

### Analysis of respondents' drinking habits

The vast majority of the responding students (n = 1186, 91%) reported that they had consumed alcohol during the preceding three months. Of the respondents who reported alcohol consumption, 55 percent were women (n = 653) and 45 percent men (n = 533).

### Self-reported weekly consumption

The self-reported mean weekly consumption for male respondents was about twice as high as the female respondents' (128 grams of alcohol for males, versus 66 grams of alcohol for females). The median values for self-reported weekly consumption followed a similar pattern (with the median value for males at 108 grams, compared to 60 grams for females).

### Heavy episodic drinking by respondents, HED

The most common binge drinking pattern for females, according to respondents, was at least once a month but less than once a week. (See Table 2.) The respondents most likely to report this pattern were female students in the age group 21–25 years (56.6%). For males, the most commonly reported binge drinking behaviour was binge drinking at least once a week. This pattern was most frequently reported by male students in the age group 21–25 years (42.9%).

**Table 2:** Frequency of occasions with heavy episodic drinking (HED) defined as 4/5 drinks or more on one occasion, n = 1186.

		HED				
			
Age	Gender	Never	Less than once a month	At least once a month and less than once a week	At least once a week	No. of participants
					
		%	%	%	%	
			
18–20 years	Male	6,3	9,8	50,9	33,0	112
	Female	14,7	14,7	50,0	20,6	136
21–25 years	Male	4,5	9,1	43,5	42,9	375
	Female	8,7	12,0	56,6	22,7	401
26–30 years	Male	13,3	20,0	46,7	20,0	30
	Female	25,9	24,1	38,9	11,1	54
31–35 years	Male	0	28,6	42,9	28,6	7
	Female	21,7	39,1	26,1	13,0	23
> 35 years	Male	22,2	44,4	22,2	11,1	9
	Female	43,6	30,8	17,9	7,7	39
						
All ages	Male	5,6	10,7	44,8	38,8	533
	Female	13,9	15,6	50,4	20,1	653

### Estimated peak blood alcohol concentration, EBAC

**Figure 1:**
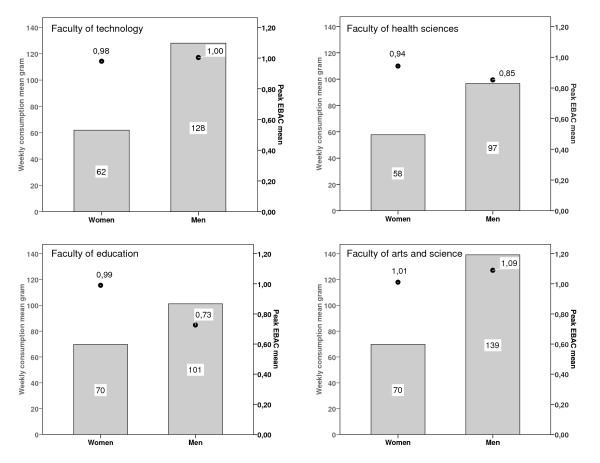
**Weekly alcohol consumption (gram) and peak EBAC (mean) distributed on gender and faculty, n = 1186**. This figure shows that men who drank alcohol reported twice the weekly consumption compared to women who drank. However, mean peak EBAC was about the same for men and women, at about 1 permillage. Men at the faculty of Arts and Science had the highest weekly consumption (139 grams/week) and the highest peak EBAC (1.09 permillage).

Men who drank alcohol reported twice the weekly consumption compared to women who drank. However, mean peak EBAC was about the same for men and women, at about 1 permillage. Men at the Faculty of Arts and Science had the highest weekly consumption (139 grams/week) and the highest peak EBAC (1.09 permillage) (Figure 1.)

### Respondents' perceptions of their own alcohol consumption in relation to consumption by other students

The responses for items measuring normative beliefs, i.e. *how much do you think that you drink compared to other students?*, were analysed in combination with stated alcohol consumption, expressed here as low or high risk. (See Table 3.) Seventy-five percent of the high-risk drinkers believed that they drank the same amount as, or less than, others.

**Table 3:** Alcohol consumption patterns by self-perceived drinking levels of respondent, comparing self to others, n = 1193.

	Low risk	High risk	Total
I do not know	16 (2%)	1 (0%)	17 (1%)
Less than others	619 (65%)	19 (8%)	638 (53%)
The same as others	283 (30%)	166 (67%)	449 (38%)
More than others	27 (3%)	62 (25%)	89 (8%)
Total	945 (100%)	248 (100%)	1193 (100%)

Alcohol consumption pattern by self-perceived drinking levels of respondent, comparing self to others, n = 1193. Respondents' perceptions of their own alcohol consumption in relation to consumption by other students. The responses for items measuring normative beliefs, i.e. *how much do you think that you drink compared to other students?*, were analysed in combination with stated alcohol consumption, expressed here as low or high risk.

### Respondents' reports of alcohol-related beliefs and negative experiences related to alcohol consumption

Several items in the e-SBI assessed respondents' agreement with beliefs that might contribute to increased alcohol intake. Other statements asked respondents to report on negative consequences of alcohol intake, related to the individual respondents, and to others in the respondent's social networks.

### Beliefs that might promote increased alcohol intake in the respondent population

Respondents were asked to agree, or disagree, with three statements related to beliefs that could promote increased alcohol intake, including: 1. Drinking makes celebrations better; 2. Drinking is a good reward for working hard; and 3. Drinking gives me better self-confidence. (See Table 4.) The vast majority of respondents agreed that alcohol improves celebrations (92%). There were significant gender differences about this belief and the other two statements, however, with males significantly more likely to agree with these statements.

**Table 4:** Statements describing student views on alcohol related social consequences, men n = 533, women n = 653.

	Respondents agreeing to each statement		
	Women %	Men %	p-value*
	
Drinking makes celebration better	90	95	< 0.001
Drinking is a good reward for working hard	45	68	< 0.001
Drinking gives me better self-confidence	67	80	< 0.001

*p-values computed for differences in gender proportions

Statements describing student views on alcohol related social consequences, men n = 533, women n = 653. Respondents were asked to agree, or disagree, with three statements related to beliefs that could promote increased alcohol intake, including: 1. Drinking makes celebrations better; 2. Drinking is a good reward for working hard; and 3. Drinking gives me better self-confidence.

### Negative experiences occurring to individual respondents

In Table 5, findings regarding reported negative consequences due to alcohol consumption are presented. The findings are adjusted for gender and academic discipline. High-risk respondents more often report problems in all categories except 'relations with family and friends'.

**Table 5:** Respondents reporting negative personal consequences due to weekly alcohol consumption, n = 1186.

	OR* High risk n = 248/Low risk n = 938	95% CI	p-value
Studies did not go well	4.3	2.6–7.2	< 0.001
Personal economy suffered	4.5	2.9–6.9	< 0.001
Negative impact on relations with family and friends	1.5	0.6–3.9	0.376
Feelings of remorse	1.9	1.2–2.9	0.004
Disturbed sleep	3.5	2.1–5.8	< 0.001
Mental health	2.3	1.2–4.4	0.016
Physical injury	2.9	1.3–3.8	0.005
Violent behaviour	4.7	1.93–11.4	0.001

*adjusted by gender and academic discipline

Respondents reporting negative personal consequences due to weekly alcohol consumption, n = 1186. In Table 5, findings regarding reported negative consequences due to alcohol consumption are presented. The findings are adjusted by gender and academic discipline.

Information describing student views on alcohol related social consequences was analysed by gender. A two-tailed z-test was used to analyse differences in proportions at a significance level of 5%.

### Negative experiences due to alcohol use by other students

Answers describing the role that alcohol plays in settings where the respondents encounter other students (see table 6), analysed by gender, reveal several differences between men and women. Significantly more women reported violence and accidents at parties and that their sleep often was disturbed due to others partying. No significant gender differences were noted for the following statements: There is often violence at parties; Property is often destroyed at parties; I often babysit drunken friends; My study habits are often disturbed due to others partying. In addition, most respondents reported ability to discuss a friends' problem drinking with him or her (90%).

**Table 6:** Statements describing student views on alcohol-related social consequences, men n = 533, women n = 653.

	Respondents agreeing to each statement		
	Women %	Men %	p-value*
	
There is often violence at parties	25	14	< 0.001
Property is often destroyed at parties	43	39	0.08
There are often accidents with physical injury at parties	54	46	0.008
I often baby-sit drunk friends	80	83	0.09
I feel I can talk to a friend about his/her alcohol habits	89	90	0.26
My sleep is often disturbed due to others partying	29	21	0.001
My study habits are often disturbed due to others partying	17	15	0.15

*p-values computed for differences in gender proportions

## Discussion

Our research provides much-needed evidence that increased consumption of alcohol is taking place in student populations, mirroring general trends in Swedish society. Overall, alcohol consumption in Sweden has increased sharply since the nation joined the European Union in 1995. Average annual alcohol consumption for people over 15 years of age, as measured in pure alcohol, grew from 8,8 litres in 1996 to 10,3 litres in 2003. Average annual alcohol consumption has since stabilised at about 10 litres per person [17,18]. Findings indicate that more Swedes are drinking regularly, and more are binge drinking. According to the Swedish Institute of Public Health, the number of binge drinking events has increased approximately 40% from 1998 to 2004 [19]. Recent research describing increased drinking in university populations stated that there is "strong evidence that frequent risk drinking is linked with the development of many different types of harm" [2]. Of particular concern are recent surveys by the Institute showing that consumption has increased amongst Swedish females ages 16–29, many of whom will be attending university [19]. Obtaining clear and current information on drinking patterns demonstrated by university students can help public health officials, university administration, and local health care providers develop appropriate prevention and treatment strategies.

Analysis of drinking habits in this university population, including self-reported weekly consumption, heavy-episodic drinking, and estimated peak blood alcohol concentration (EBAC), indicate that some of the respondents in this study are experiencing problems, due to their own or others' drinking behaviours. Respondents who reported high risk weekly consumption (n = 248) more often report problems in all categories except 'relations with family and friends'. For example, high-risk drinkers were significantly more likely to report that they had experienced or witnessed violent behaviour (OR 4.7, p value < 0.001), had bad economic consequences (OR 4.5, p value < 0.001), and experienced bad results in their studies (OR 4.3, p value < 0.001), as a result of their drinking, compared to low-risk drinkers. (See Table 5.) This awareness of the negative consequences of excess alcohol intake by high-risk drinkers may be a factor that can help student health workers build effective intervention strategies.

Analysis of feedback related to respondents' perceptions of their own alcohol consumption in relation to consumption by other students, (i.e. normative beliefs) revealed that about seventy-five percent of the high-risk drinkers believed that they consumed about the same amount, or less, alcohol than their peers. This finding reveals that most high-risk drinkers in our study population were not aware of normal consumption patterns for university students. Other studies have suggested that providing feedback about normative behaviour, as was done with this project, can help high-risk students more accurately judge and adjust their own intake [20,21]. Many university students tend to over-estimate their peers' alcohol consumption [10]. If this tendency to overestimate others' intake contributes to high-risk drinkers' intakes, as has been suggested, providing personalized feedback in which normative misperceptions are corrected may reduce heavy episodic drinking [22]. Follow up studies to this survey will help assess whether normative feedback has been effective in reducing alcohol consumption amongst high-risk drinkers in our own study population.

Our study analyzed respondents' beliefs about alcohol use and negative experiences related to alcohol consumption, either by the individual student respondents or other students, and found that the vast majority (92% of respondents) agreed with the statement that alcohol improves celebrations. On the other hand, the majority of students noted multiple circumstances in which other students' drinking impacted their own quality of life, including sleep disturbances, exposure to violence at parties, and having to babysit drunken friends. Given that alcohol appears to be 'required' for celebrations, future efforts may be best directed toward decreasing consumption during these events, so that negative consequences can be avoided. Increasing awareness about the negative impact of one's excess alcohol consumption on one's peers, i.e., needing to be babysat by friends, may also prove an effective approach in decreasing alcohol intake.

Overall, our cooperation with Student Health Services in developing and implementing this survey was quite helpful. For respondents, a link to the Services homepage was provided to allow students to access information immediately upon completing surveys. In addition, a touch screen computer was available for students who were visiting the Student Health Services, accessing the same sort of survey and feedback used in this project. Personnel at Student Health Services were aware of every phase of this study, and were prepared to offer support for students who appeared at the clinic after taking the survey who wished help with decreasing alcohol consumption.

### Study Limitations

Our findings have limitations. The response rate to the survey was about 46%, a fairly high response rate for an unsolicited survey request, but not a complete census. No incentives were provided to respondents, which may have otherwise increased participation. It is possible that many potential respondents overlooked their email invitations-subsequent surveys might experience increased response rates by providing a link to the survey via the university's home page. The survey was limited to students who could read Swedish. Self-reported answers were not verified by other information sources. We note that for any survey for which participation is voluntary, it is possible that the responses from participants are not reflective of the entire study population. We note also that gender balance in responses is not evenly balanced, when viewed by academic discipline. This is partially due to the gender make up of the various disciplines – in particular; males are over-represented in technological studies, while there are more females enrolled in education and health sciences. We also note that the generalisability of this study must be considered against other factors that impact drinking behaviour in Sweden, in particular the national policy to limit access to alcohol via higher prices and limited hours for purchase of alcohol via state-run stores [23].

## Conclusion

The use of computerised surveys to provide alcohol screening in university populations is effective on many levels. Large numbers of students can be screened simultaneously, and at low cost. Information on student alcohol consumption habits can be quickly obtained, and interventions designed based on respondents' behaviours, beliefs, and the university environment in which they live.

Our study revealed that the use of a Swedish-language e-SBI can provide the advantages listed above, for use in future treatment and prevention campaigns in this country. The results of the survey dispelled several assumptions that had been made about our study populations. Specifically, we found that when women drink to intoxication levels, they reach the same permillage alcohol content as men. In summary, this study suggests new evidence about drinking patterns in university students in Sweden. The study shows that high risk drinkers tend to underestimate their own consumption compared to others, and that these high risk drinkers experience more negative consequences after alcohol intake, than other respondents. There was a strong belief, for both high- and low-risk drinkers, that alcohol helped celebrations be more festive. This study also confirms findings from other study locations that while males drank more than females in our study population, females reached the same peak alcohol blood concentrations as males. Obtaining clear and current information on drinking patterns demonstrated by university students, such as was done in this study, can help public health officials, university administration, and local health care providers develop appropriate prevention and treatment strategies.

## Competing interests

The authors declare that they have no competing interests.

## Authors' contributions

Authors contributing to this paper included: AA, primary investigator, co-author of all drafts of this paper, assisted in statistical analysis, study design. A-BW, statistical analysis. CÖ, study design, support for web-based survey and response storage. PB, original research idea and DSE, co-author, all drafts. All authors read and approved the final manuscript.

## Pre-publication history

The pre-publication history for this paper can be accessed here:


